# Cathelicidin Host Defence Peptide Augments Clearance of Pulmonary *Pseudomonas aeruginosa* Infection by Its Influence on Neutrophil Function *In Vivo*

**DOI:** 10.1371/journal.pone.0099029

**Published:** 2014-06-02

**Authors:** Paula E. Beaumont, Brian McHugh, Emily Gwyer Findlay, Annie Mackellar, Karen J. Mackenzie, Richard L. Gallo, John R. W. Govan, A. John Simpson, Donald J. Davidson

**Affiliations:** 1 MRC Centre for Inflammation Research, Queen's Medical Research Institute, The University of Edinburgh, Edinburgh, United Kingdom; 2 Division of Dermatology, Department of Medicine, University of California at San Diego and VA San Diego Health Care System, San Diego, California, United States of America; 3 Edinburgh Infectious Diseases, The Chancellor's Building, New Royal Infirmary, University of Edinburgh, Edinburgh, Scotland, United Kingdom; 4 Institute of Cellular Medicine, Medical School, Newcastle University, Newcastle upon Tyne, United Kingdom; Louisiana State University, United States of America

## Abstract

Cathelicidins are multifunctional cationic host-defence peptides (CHDP; also known as antimicrobial peptides) and an important component of innate host defence against infection. In addition to microbicidal potential, these peptides have properties with the capacity to modulate inflammation and immunity. However, the extent to which such properties play a significant role during infection *in vivo* has remained unclear. A murine model of acute *P. aeruginosa* lung infection was utilised, demonstrating cathelicidin-mediated enhancement of bacterial clearance *in vivo*. The delivery of exogenous synthetic human cathelicidin LL-37 was found to enhance a protective pro-inflammatory response to infection, effectively promoting bacterial clearance from the lung in the absence of direct microbicidal activity, with an enhanced early neutrophil response that required both infection and peptide exposure and was independent of native cathelicidin production. Furthermore, although cathelicidin-deficient mice had an intact early cellular inflammatory response, later phase neutrophil response to infection was absent in these animals, with significantly impaired clearance of *P. aeruginosa*. These findings demonstrate the importance of the modulatory properties of cathelicidins in pulmonary infection *in vivo* and highlight a key role for cathelicidins in the induction of protective pulmonary neutrophil responses, specific to the infectious milieu. In additional to their physiological roles, CHDP have been proposed as future antimicrobial therapeutics. Elucidating and utilising the modulatory properties of cathelicidins has the potential to inform the development of synthetic peptide analogues and novel therapeutic approaches based on enhancing innate host defence against infection with or without direct microbicidal targeting of pathogens.

## Introduction

Cationic host-defence peptides (CHDP; also known as antimicrobial peptides or AMPs) are important components of early innate host defences. In addition to their physiological roles, these peptides and their derivatives have been proposed as future antimicrobial therapeutics, relatively unaffected by the development of sustained microbial resistance [1]. Although initially characterised as directly microbicidal agents, it is now clear that many CHDP also have multiple functions as modulators of inflammation and immunity [2], [3], [4], with emerging roles in diseases affecting multiple organs including the lung, skin and gastrointestinal tract. Human clinical trials using analogues of CHDP modified to maximise direct microbicidal function have achieved only moderate efficacy [5], perhaps due to failure to recognise the importance of the immunomodulatory functions of the native peptides. Interestingly, studies using non-microbicidal analogues of naturally-occurring CHDP that retained other bioactive functions, have demonstrated effective host defence augmentation in mice [6], [7]. These studies raise questions about the relative roles of microbicidal and immunomodulatory properties of naturally-occurring CHDP in infections.

Cathelicidins are multipotent immunomodulatory CHDP [8]. The sole human cathelicidin Human Cationic Antimicrobial Peptide of 18 kDa (hCAP-18; encoded by *CAMP*) is expressed by multiple cell types including neutrophils, where it is stored in specific granules and proteolytically cleaved following release, to produce a 37 amino acid mature peptide fragment named LL-37 [9], [10]. hCAP-18/LL-37 is upregulated in pulmonary infections [11] and, in children with RSV bronchiolitis, low serum cathelicidin is correlated with more severe disease [12]. Mice, like humans, express only a single cathelicidin gene; *Camp* (encoding the mCRAMP peptide) [13], with similar patterns of expression, which is cleaved to produce an active 34 amino acid peptide [14]. Mice deficient in mCRAMP (*Camp*−/−) have increased susceptibility to bacterial infections of the skin [15], intestinal tract [16], cornea [17] and urinary tract [18]. These *Camp*−/− mice also have impaired host defence against lung infection [19], [20], while therapeutic use of LL-37 and/or mCRAMP in wild type mice is protective in models of pulmonary infection with *P. aeruginosa* [21] or influenza virus [22]. These studies demonstrate a critical, non-redundant role for endogenous cathelicidin in host defence against lung infection and the therapeutic potential of the unmodified peptides, but the mechanisms by which pulmonary host defence is enhanced *in vivo* remains unclear. Although generally presented as being primarily a consequence of direct microbicidal activity, this is not fully consistent with *in vivo* concentrations and microbicidal properties in a physiological environment. However, the extent to which any of the plethora of immunomodulatory properties ascribed to cathelicidins play a significant role during infection has never been demonstrated *in vivo*. Understanding the critical modulatory roles of native CHDP and how these contribute to innate host defence against infection, may prove to be vital in development of specific pathogen-targeted analogues of these peptides for therapeutic use.

Respiratory diseases are among the most common causes of morbidity and account for 1 in 5 deaths in the UK [23]. A third of mortalities are due to acute respiratory infections, influenza or pneumonia and pathogens resistant to conventional therapeutics represent an increasing clinical challenge. *Pseudomonas aeruginosa* is the primary cause of nosocomial pulmonary infections and pulmonary colonisation with this pathogen is considered to be responsible for the fatal deterioration of lung function in patients with cystic fibrosis (CF) (reviewed in [24]). This opportunistic pathogen is difficult to treat because of its widespread resistance to multiple antibiotics [25], with the limited number of effective antimicrobial treatments reduced further by the emergence of carbapenem-and polymyxin-B resistant isolates [26]. A greater understanding of the natural host defence mechanisms involved in pulmonary defence against this organism is required in order to develop novel therapeutic approaches. Cathelicidins can alter susceptibility to pulmonary infection with *P. aeruginosa* in murine models [19], [20], [21], despite this pathogen being resistant to the directly microbicidal effects of these peptides in the presence of physiologically relevant levels of cations *in vitro* [27], [28], [29]. Thus, the *in vivo* roles of endogenous cathelicidin in host defence against *P. aeruginosa*, the relative effects of microbicidal and modulatory properties, and the consequences of therapeutic targeting of cathelicidin expression or exogenous delivery of peptide remain unknown.

We demonstrate that therapeutically administered synthetic LL-37 peptide can enhance the clearance of *P. aeruginosa* from the murine lung, in the absence of demonstrable direct microbicidal effects, and can induce an upregulation of the early neutrophil response to pathogen in the lungs that is dependent both upon the presence of the peptide and the pathogen. We show that despite a normal early neutrophil response, second phase pulmonary neutrophil influx was deficient in *Camp*−/− mice, with impaired clearance of pulmonary *P. aeruginosa*. Delivery of LL-37 to these cathelicidin-deficient mice enhanced the neutrophil response and restore bacterial clearance, demonstrating proof of principle for therapeutic use of LL-37 in cathelicidin deficiency. These studies indicate that the protective effects of cathelicidins in *P. aeruginosa* infection *in vivo* can result from modulatory effects in innate immune responses, synergising with infectious stimuli to enhance a protective neutrophil response.

## Materials and Methods

### Peptide

LL-37 (LLGDFFRKSKEKIGKEFKRIVQRIKDFLRNLVPRTES; MW 4493.33) was either synthesised by N-(9-fluorenyl) methoxycarbonyl chemistry at the Nucleic Acid/Protein Service unit at the University of British Columbia (UBC; Vancouver, Canada), or custom synthesised by Almac (East Lothian, Scotland) using Fmoc solid phase synthesis and reversed phase HPLC purification. Peptide identity was confirmed by electrospray mass spectrometry, purity (>95% area) by RP-HPLC and net peptide content determined by amino acid analysis. Lyophilised peptides were reconstituted in endotoxin free water at 5 mg/ml stock concentration and determined to be endotoxin-free using a Limulus Amebocyte Lysate Chromogenic Endotoxin Quantitation Kit (Thermo Scientific, UK). Peptide functionality was confirmed by assessing anti-endotoxic activity [30].

### Bacteria

*Pseudomonas aeruginosa* strain PAO1 was grown in Luria Bertani (LB) broth at 37°C in an orbital shaker (250 rpm) overnight to achieve a stationary-phase suspension. Bacterial cultures were then diluted 1∶10 in fresh LB broth and incubated at 37°C for 90 min to reach logarithmic phase. Standardisation was performed by dilution with LB broth to an optical density of 0.1 at 595 nm using spectrophotometry (WPA UV 1101, Biotech Photometer), then bacteria were centrifuged at 1,500×g for 15 min and resuspended in PBS for use.

### Murine infection model

Mouse experiments were performed in accordance with Home Office UK project licence 60/4216, under the Animal (Scientific Procedures) Act 1986. Wild type control mice were all C57Bl/6 strain, supplied by Charles River Laboratories, UK, and housed at the University of Edinburgh for at least two weeks before use, or were animals bred from those mice. *Camp −/−* mice [15] were bred to congenicity on a C57Bl/6 strain background and were the offspring of homozygous mutant matings raised in the same facility at the University of Edinburgh. Both male and female mice were used, between 8–12 weeks old, housed in individually ventilated cages and randomly assigned to treatment groups (no significant difference were found in end points between male and female mice). Mice were weighed, given a general anaesthetic (isofluorane) in a category 2 biosafety hood, then held vertically by scruffing over the front of the thorax and inoculated by an intranasal delivery up to a total of 50 µl volume. Mice were inoculated with 3×10^7^ colony forming units (cfu) of PAO1 or the same volume of PBS, and 10 µg LL-37 peptide in PBS or PBS only control. PBS alone (carrier for both bacteria and peptide) was used as a control (rather than scrambled peptide, which previous pulmonary infection studies indicated had no effects ([22] and unpublished data), in order that the wild type control infected animals were appropriate controls both for the LL-37-treated infected wild types and for the infected *Camp−/−* animals (in which no peptide was delivered). Mice were then returned to cages, placed on a heat mat to maintain body temperature, and monitored and scored for signs of infection every 2 hours, with peak of illness occurring at 6–8 hours post infection, followed by recovery with diminishing severity score. Mice were re-weighed and culled at selected timepoints (0, 6 or 24 hours), culled by pentobarbital injection and lungs and trachea exposed by dissection. Lungs were lavaged in 1 ml sterile PBS via intramedic polyethylene tubing (Sigma Aldrich, UK) inserted into the trachea, and bronchoalveolar lavage fluid (BALF) stored on ice. Following lavage, lungs were either homogenised in 2 ml sterile PBS for cfu counts or were perfused by PBS injection into the heart, then removed and frozen for RNA preparation.

### CFU counts

BALF or homogenised lungs were serially diluted in PBS, plated on *Pseudomonas* Isolation agar (Becton Dickinson Difco, Oxford, UK), incubated overnight at 37°C and bacterial colonies counted using a Stuart SC6 colony counter. Total colonies on the lowest dilution plate countable were multiplied by the appropriate dilution factors to determine the total CFU count of the lung tissue or BALF sample.

### Cytospins and counts

BALF was centrifuged at 200×g for 5 minutes, and supernatant was removed for cytokine measurement. Pelleted cells were resuspended and counted by NucleoCounter YC-100 (ChemoMetec, Allerød, Denmark) automated cell number counting. 100 µl of cell suspension was then loaded onto a glass slide using a disposable sample funnel and cytocentrifuged at 10×g for 3 minutes in a Shandon Cytospin 2 centrifuge. Slides were air dried for 20 minutes, fixed in methanol for 20 minutes, stained with Diff Quik (Fisher Scientific, Loughborough, UK), and mounted in DPX Mountant (Fluka BioChemika/Sigma Aldrich, UK). Differential counts for neutrophils and monocytes were then performed by light microscopy at 20× magnification using an EVOS FL microscope (Peqlab, Sarisbury Green, UK).

### ELISAs

BALF was used to measure cytokine levels by ELISA according to manufacturer's instructions, for KC, MIP-2 alpha (R&D Systems, UK) or using a cytometric bead assay mouse inflammation kit (BD Biosciences, UK) for TNF, IL-6, MCP-1, IL-10, IFN-gamma, IL-12p70.

### qRT-PCR

Mouse lung tissue was homogenized in Qiagen RLT buffer (Qiagen, Manchester, UK) using a Precellys 24 homogeniser with Precellys-Keramik-kit ceramic beads (PeqLab). RNA was then prepared from homogenised mouse lung tissue using RNeasy mini kits (Qiagen), according to the manufacturer's instructions. After DNase treatment with RQ1 DNase (Promega, Southampton, UK), cDNA was prepared from RNA using TaqMan reverse transcriptase reagents and random hexamer primers (Life Technologies Ltd, Paisley, UK), according to the manufacturer's instructions. Quantitative Real Time PCR was performed on a StepOne Real Time PCR machine (Life Technologies), using Gene Expression Mastermix and TaqMan gene expression assays for *Camp* (assay I.D. Mm00438285_m1) and 18S (assay I.D. Mm03928990_g1). Relative quantitation of *Camp* was calculated using the ΔCT method.

### Analysis of mCRAMP protein expression

Harvested lung tissue was placed in 600 µl M-PER Mammalian Protein Extraction Reagent with Complete Protease Inhibitor Cocktail (Roche Applied Science, Burgess Hill, UK) added, and homogenised using a Precellys 24 homogeniser with Precellys-Keramik-kit ceramic beads (PeqLab). Homogenised tissue was shaken on an IKA-Vibramax-VXR (Sigma Aldrich, UK)) for 20 minutes at 4°C and lysates were subsequently centrifuged at 15,000×g for 10 minutes at 4°C to pellet insoluble material. Protein concentration in lysates was measured by Pierce BCA assay (Thermo Scientific), according to manufacturer's instructions. Lysate concentrations were equalised with lysis buffer, and subsequently boiled at 96°C for 5 minutes in the presence of loading buffer and reducing agent (Life Technologies), then run on Novex NuPAGE 4–12% Bis-Tris pre-cast gels, in MOPS buffer (Life Technologies), and subsequently transferred to Novex 0.2 µm pore Nitrocellulose Membrane. mCRAMP was detected with rabbit anti-mouse mCRAMP antibody (R-170, Santa Cruz Biotechnology, Heidelberg, Germany), followed by staining with IRDye 800CW anti-rabbit secondary antibody, with subsequent detection using a LI-COR Odyssey Infrared Imaging System. Rabbit pan-actin antibody (Cell Signalling Technology, Danvers, MA, USA) staining was used as a loading control, detected with anti-rabbit secondary antibody and infrared imaging as above. Mouse CRAMP staining was then quantitated using LI-COR Odyssey software.

### Statistics

Statistical analyses were performed using Graphpad Prism version 5.04 for Windows. Bacterial counts were normalised by logarithmic transformation before analysis by 2 way ANOVA with Bonferroni's post tests where appropriate. Cell counts and cytokine concentrations were analysed by Mann Whitney test. Differences were considered statistically significant at P<0.05.

## Results

### LL-37-mediated microbicidal activity against *P. aeruginosa* PAO1 is not observed *in vivo*

**Figure 1:**
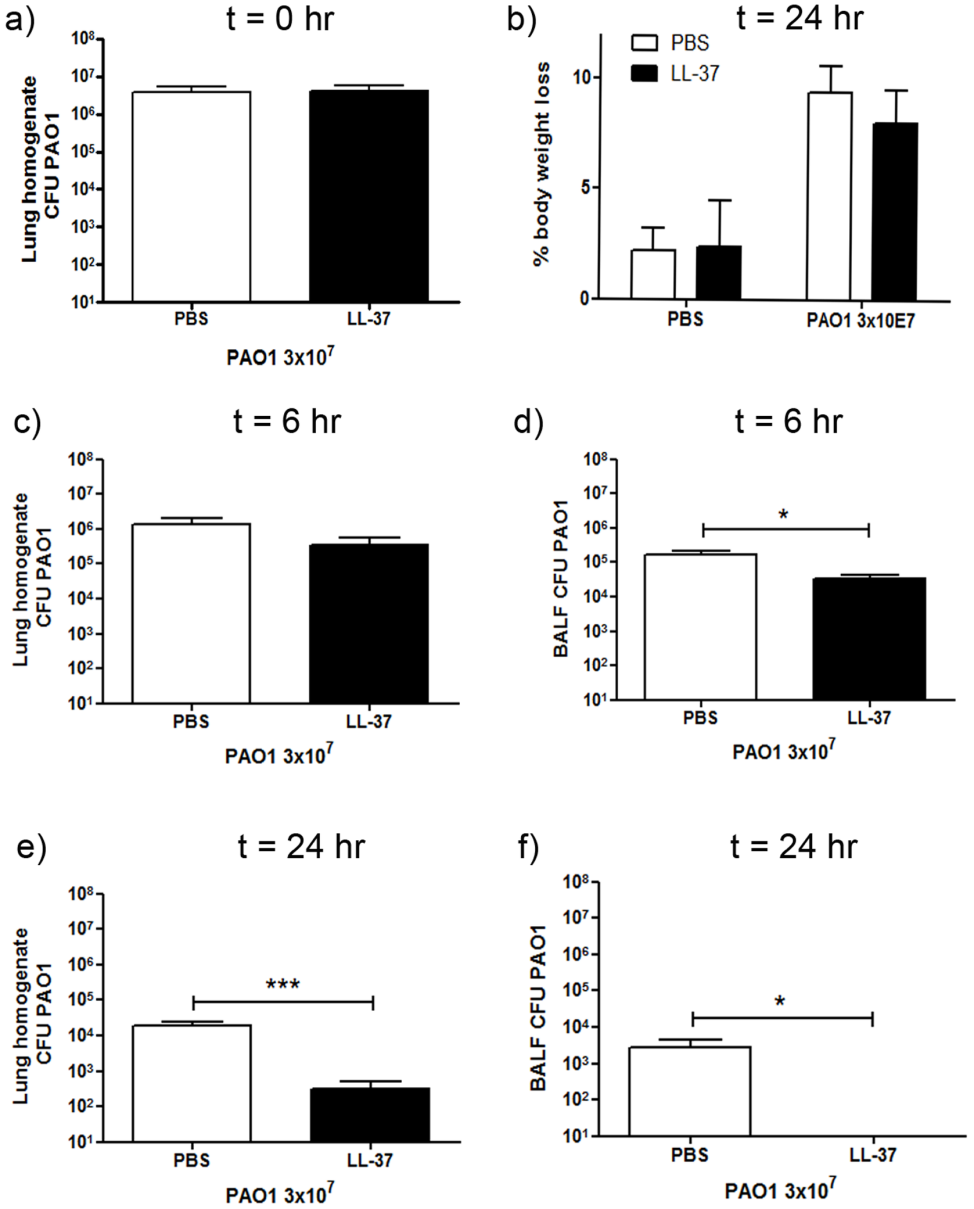
Exogenous LL-37 enhances pulmonary clearance of *P. aeruginosa*. Wild type C57Bl/6 mice were weighed, then inoculated with 3×10^7^ cfu of *P. aeruginosa* PAO1 or PBS and 10 µg LL-37 peptide or PBS by intranasal delivery. a) Immediately after inoculation of all mice, a subset (called 0 hr; n = 3 per group) were culled and their lungs homogenised (60 minutes after initial inoculation), or b–f) 6 or 24 hours after inoculation mice were re-weighed and culled, and their lungs were lavaged before homogenisation. BALF and lung homogenates were serially diluted, plated and incubated overnight at 37°C before bacterial colonies were counted and corrected for volume. Mean PAO1 cfu +/− SEM in the lung homogenate (a, c & e) or BALF (d & f) for infected animals (n≥9 per condition) are displayed. No bacteria were detected in samples from uninfected mice. b) Data show mean percentage weight loss +/− SEM. For statistical analyses bacterial counts were normalised by logarithmic transformation. Analyses were conducted using 2 way ANOVA with Bonferroni's post tests; * p<0.05, ** p<0.01.

LL-37 and other CHDP were initially described as having rapid direct microbicidal properties based on *in vitro* studies conducted under favourable ionic environments (reviewed in [27]). However, the activity of LL-37 can be inhibited by physiological levels of divalent cations [27], [28], serum apolipoprotein, f-actin and DNA [31], [32], [33]. Thus, the relative contributions of microbicidal versus modulatory properties in conferring the protective effects of this peptide *in vivo* remain uncertain. We and others have shown that LL-37 has negligible microbicidal activity against *P. aeruginosa in vitro* [27], [28], [29]. In order to evaluate the contribution of any early direct microbicidal properties of exogenously delivered LL-37 peptide in a murine pulmonary *P. aeruginosa* infection model, mice were culled immediately after the intranasal delivery of bacteria with peptide or carrier-only control to the cohort. Lungs were removed, homogenised and plated to evaluate the number of viable bacteria in the lungs. These samples are referred to as t = 0, however homogenisation did not occur until 60 minutes after inoculation, during which time interaction between peptide and bacteria could occur. No significant difference was observed between infected mice receiving LL-37 and control infected animals (Fig 1a) demonstrating that LL-37, under these conditions, had no discernable early microbicidal affects.

### Therapeutic delivery of LL-37 is protective against *P. aeruginosa* infection *in vivo*

In order to evaluate the protective antimicrobial properties of LL-37 against *P. aeruginosa* PAO1 *in vivo*, mice were infected with or without concomitant delivery of LL-37. All mice lost ∼4% body weight in the first 6 hours post procedure (data not shown), with infected animals continuing to lose weight over 24 hours, but no significant effect of LL-37 treatment was observed (Fig 1b). Mice were culled 6 and 24 hours post-infection and the total number of viable bacteria in the BALF (bronchoalveolar lavage fluid) and lung homogenate was assessed (Fig 1 c–f). At 6 hours post-infection, treatment with LL-37 showed no statistically significant effect on the total number of bacteria in the lung homogenates, but did result in significantly lower levels of bacteria in the BALF (Fig 1c/d). By 24 hours post-infection, LL-37 treatment had significantly enhanced pathogen clearance from the lungs, compared to controls, leaving only a residual infection, inaccessible to BAL (Fig 1e/f). These data demonstrate the capacity of LL-37 to enhance pulmonary bacterial clearance in the absence of early microbicidal properties.

### Therapeutic delivery of LL-37 enhances neutrophil responses in infected animals

**Figure 2:**
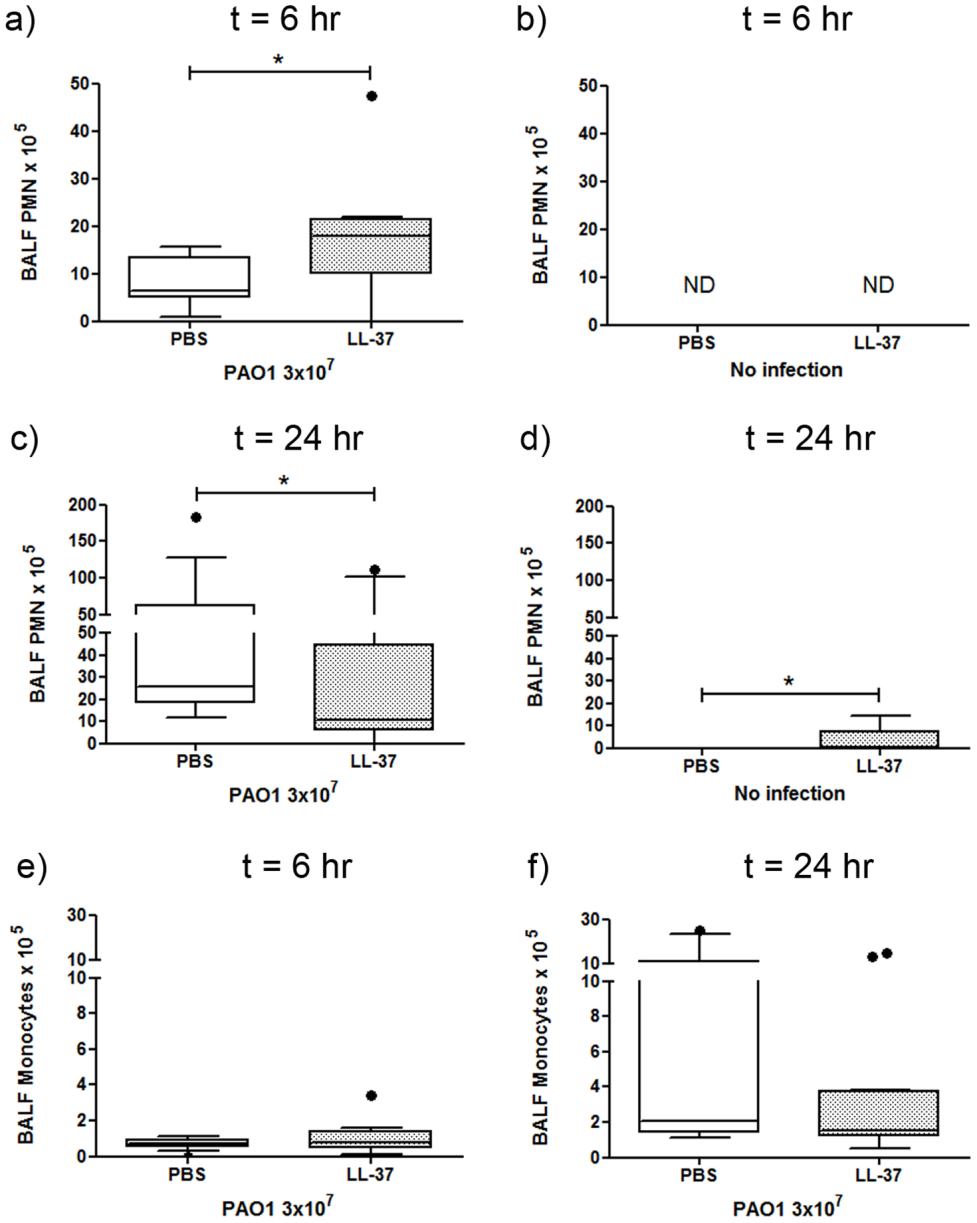
Exogenous LL-37 promotes an early neutrophil response to *P. aeruginosa*. Wild type C57Bl/6 mice were inoculated with 3×10^7^ cfu of *P. aeruginosa* PAO1 or PBS and 10 µg LL-37 peptide or PBS by intranasal delivery. At 6 hours (a, b & e) or 24 hours (c, d & f) after inoculation mice were culled and their lungs were lavaged. BALF was cytocentrifuged and differential counts were conducted for neutrophils (a–d) and monocytes (e & f). Data show Tukey box and whiskers plots for infected (a, c, e & f) (n≥9 per condition) and uninfected (b & d) animals (n≥5 per condition). Analyses were conducted using the Mann Whitney test; * p<0.05. ND denotes “not detected”.

Cathelicidins have been proposed to have multiple inflammomodulatory properties that could modulate the clearance of infection *in vivo* [2], including direct chemotactic activity of LL-37 for neutrophils and monocytes [34], [35], [36], [37], [38]. Differential cytospin cell counts were performed on the BALF from LL-37-treated and control-treated infected and uninfected mice at 6 and 24 hours after infection. LL-37 treatment resulted in a significantly upregulated neutrophil response to infection (over 2 fold increase in median cell number) compared to control infected animals at 6 hours post-infection (Fig 2a). No neutrophils were detected in the BALF from uninfected mice, regardless of LL-37 treatment, at this timepoint (Fig 2b). Whereas a larger second phase neutrophil response was observed by 24 hours in control-treated infected mice, the mean neutrophil number in the LL-37-treated infected animals was actually significantly lower than the controls at this timepoint, in line with the enhanced earlier clearance of the infection (Fig 2c). A degree of neutrophil influx was observed in response to LL-37 alone at 24 hours in uninfected mice (Fig 2d). In contrast, no significant LL-37-mediated effects on pulmonary monocyte numbers were observed at 6 or 24 hours in infected (Fig 2 e/f) or uninfected (data not shown) mice.

### Therapeutic delivery of LL-37 does not affect pulmonary cytokine responses in infected animals

**Figure 3:**
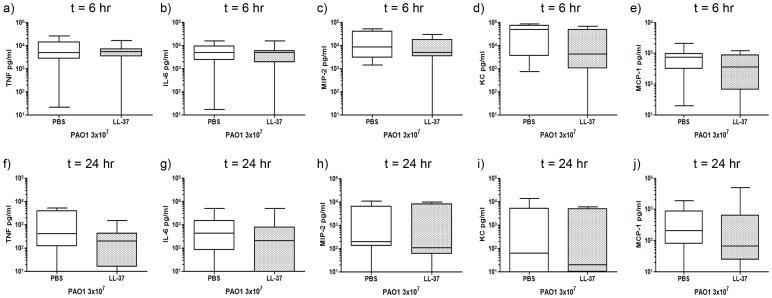
*P. aeruginosa*, but not exogenous LL-37, induces pulmonary cytokine responses. Wild type C57Bl/6 mice were inoculated with 3×10^7^ cfu of *P. aeruginosa* PAO1 and 10 µg LL-37 peptide or PBS by intranasal delivery. At 6 hours (a–e) or 24 hours (b–j) after inoculation, mice were culled and their lungs were lavaged. BALF was centrifuged to remove cells and levels of TNF (a, f), IL-6 (b, g), MIP-2 (c, h), KC (d, i) and MCP-1 (e, j) were determined. Data show Tukey box and whiskers plots for n≥9 animals per condition. Analyses were conducted using the Mann Whitney test.

In order to determine whether LL-37-mediated enhanced neutrophil responses were secondary to modulation of pulmonary cytokine and chemokine responses, CBA (Cytometric Bead Array) and ELISA assays were performed on the BALF from LL-37-treated and control-treated infected mice at 6 and 24 hours after infection, to determine the concentrations of TNF, IL-6, MIP-2, KC, MCP-1, IL-10, IFNγ and IL-12. Although TNF, IL-6, MIP-2, KC and MCP-1 were all highly expressed in response to infection at 6 hours (Fig 3a–e) compared to baseline levels in uninfected mice (data not shown), and resolving by 24 hours (Fig 3f–j), treatment with LL-37 had no significant effect on any of the cytokines measured. In contrast, IL-10, IFNγ and IL-12 were not detected in significant quantities.

### Pulmonary infection with *P. aeruginosa* induces *Camp* expression in the murine lung

In this model, therapeutic administration of LL-37 was in addition to any effects of endogenous murine cathelicidin mCRAMP produced in the murine lungs in response to infection. mCRAMP has also been shown to have neutrophil chemotactic properties *in vivo*, in an air pouch model [36]. Thus, in order to establish the temporal expression pattern of *Camp* in *P. aeruginosa* infected mice, qRT-PCR and western immunoblot analyses were performed on lung homogenates at 0, 2, 6 and 24 hours post-infection. *Camp* transcription was not detected at 0 hour, but was detectable at very low levels by 2 hours after infection. Transcription was dramatically increased at 6 and 24 hours after infection (upregulated 1886-fold, +/−137, and 1124-fold, +/−66 respectively, relative to the 2 hour timepoint), with mCRAMP protein clearly detectable at these timepoints (data not shown) in keeping with previously published data [20]. Thus, the inflammatory responses were potentially modified by cathelicidin from around 6 hours post-infection in all mice, but additionally modified by cathelicidin within the first few hours in mice receiving an intranasal bolus of LL-37.

### Endogenous mCRAMP is protective against *P. aeruginosa* infection *in vivo*

**Figure 4:**
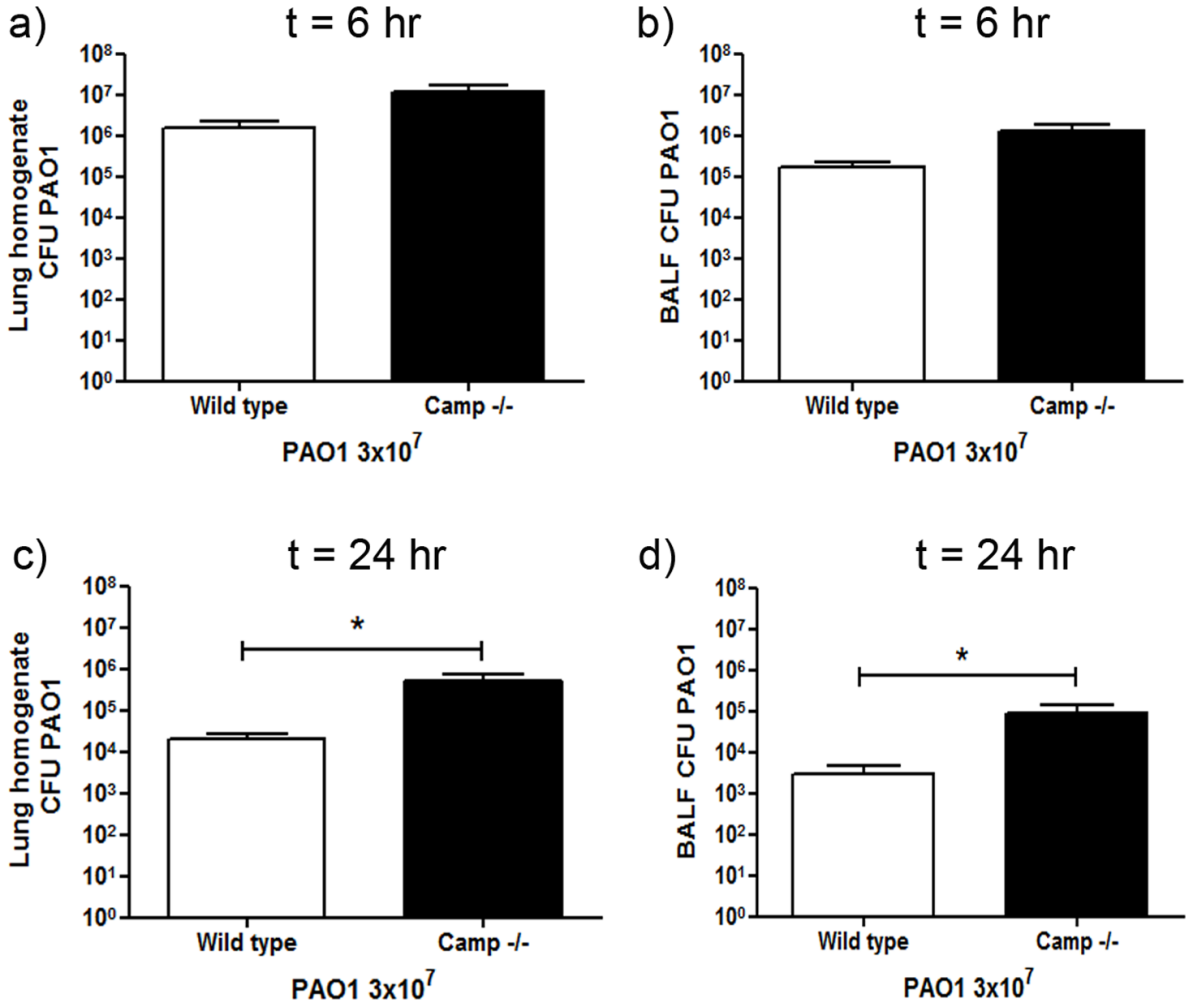
Cathelicidin-deficient mice display impaired pulmonary clearance of *P. aeruginosa*. *Camp−/−* mice and wild type controls were inoculated with 3×10^7^ cfu of *P. aeruginosa* PAO1 or PBS by intranasal delivery. At 6 or 24 hours after inoculation mice were culled and their lungs were lavaged before homogenisation. BALF and lung homogenates were serially diluted, plated and incubated overnight at 37°C before bacterial colonies were counted and corrected for volume. Mean PAO1 cfu +/− SEM in the lung homogenate (a & c) or BALF (b & d) for infected animals (n≥10 per condition) are displayed. No bacteria were detected in samples from uninfected mice. For statistical analyses bacterial counts were normalised by logarithmic transformation. Analyses were conducted using 2 way ANOVA with Bonferroni's post tests; * p<0.05.

In order to evaluate the protective antimicrobial properties of induced endogenous mCRAMP against *P. aeruginosa* PAO1 in this model, *Camp −/−* mice were infected and compared to wild type control animals. The profile of weight loss in infected *Camp −/−* mice was not significantly different from the wild type control (data not shown). Mice were culled 6 and 24 hours post-infection and the total number of viable bacteria in the BALF and lung homogenate was assessed (Fig 4 a–d). At 6 hours post-infection *Camp −/−* mice showed no statistically significant difference in the total number of bacteria in the lung homogenates or BALF when compared to infected wild type controls (Fig 4 a/b). However, by 24 hours post-infection, wild type mice had more effectively cleared the bacteria, with significantly higher pathogen loads found in both the lung homogenates and BALF from the *Camp −/−* mice (Fig 4c/d). These data demonstrate the capacity of endogenous mCRAMP to enhance pulmonary bacterial clearance, occurring after inducible *Camp* expression is detectable in the lungs of wild-type mice.

### Endogenous mCRAMP enhances neutrophil responses in infected animals

**Figure 5:**
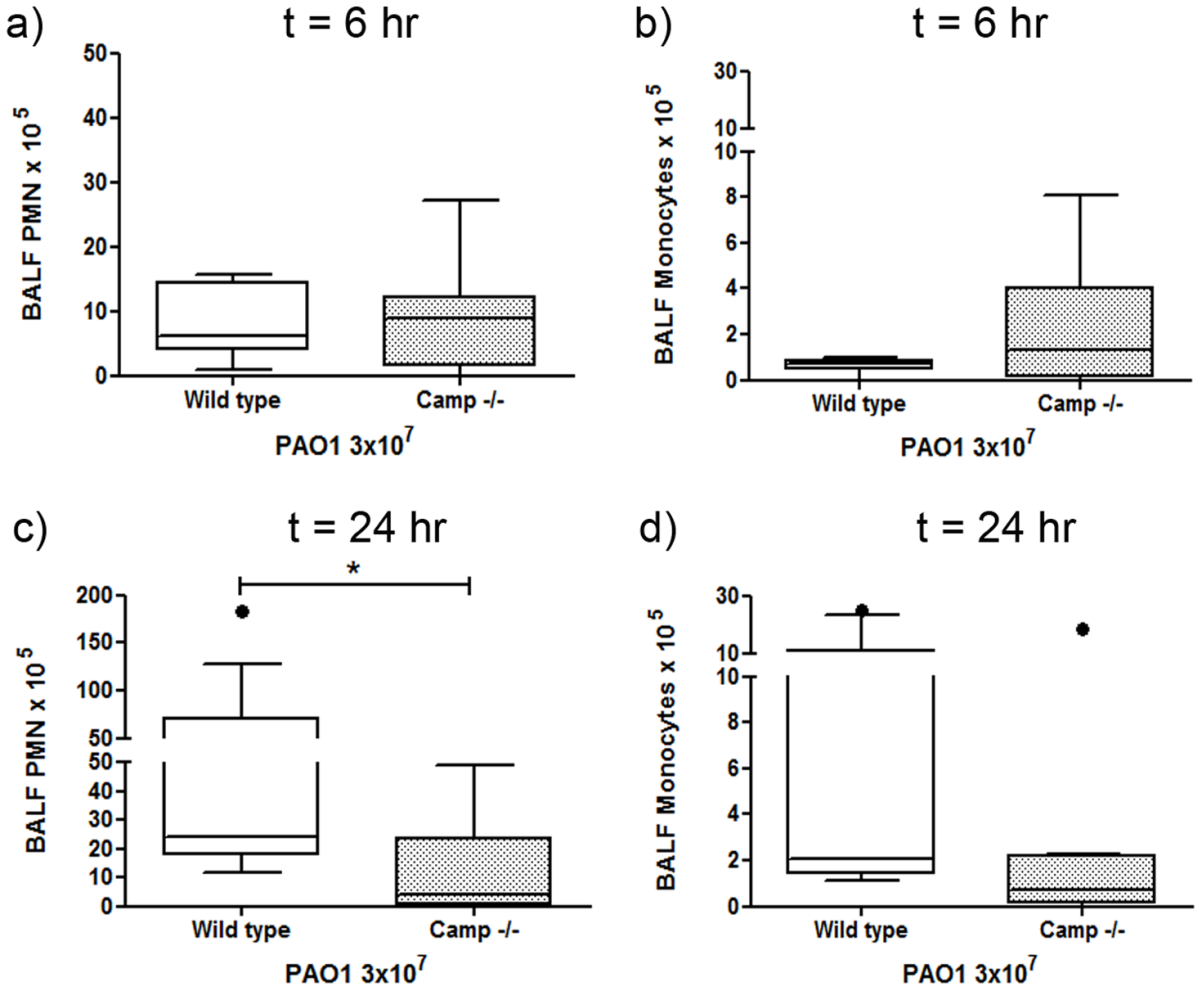
Cathelicidin-deficient mice display impaired late neutrophil responses to *P. aeruginosa*. *Camp−/−* mice and wild type controls were inoculated with 3×10^7^ cfu of *P. aeruginosa* PAO1 by intranasal delivery. At 6 hours (a & b) or 24 hours (c & d) after inoculation mice were culled and their lungs were lavaged. BALF was cytocentrifuged and differential counts were conducted for neutrophils (a & c) and monocytes (b & d). Data show Tukey box and whiskers plots for n≥8 animals per condition. Analyses were conducted using the Mann Whitney test; * p<0.05.

**Figure 6:**
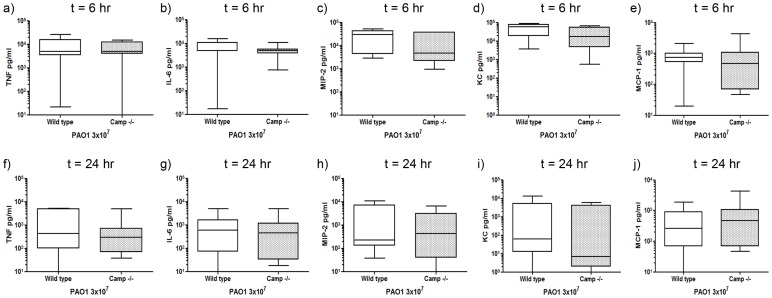
*P. aeruginosa*, but not cathelicidin sufficiency, induces pulmonary cytokine responses. *Camp−/−* mice and wild type controls were inoculated with 3×10^7^ cfu of *P. aeruginosa* PAO1 by intranasal delivery. At 6 hours (a–e) or 24 hours (b–j) after inoculation, mice were culled and their lungs were lavaged. BALF was centrifuged to remove cells and levels of TNF (a, f), IL-6 (b, g), MIP-2 (c, h), KC (d, i) and MCP-1 (e, j) were determined. Data show Tukey box and whiskers plots for n≥8 animals per condition. Analyses were conducted using the Mann Whitney test.

The extent to which endogenous mCRAMP might play a role in the pulmonary neutrophil response to *P. aeruginosa* infection was examined by comparing BALF cytospin differential cell counts from infected *Camp −/−* mice and wild type mice at 6 and 24 hours after infection. No significant differences were observed in neutrophil or monocyte counts at 6 hours (Fig 5a/b), with an initial neutrophil influx occurring similarly in both genotypes (Fig 5a). However, *Camp −/−* mice failed to further upregulate this response, demonstrating a significantly less elevated neutrophil count than wild type controls at 24 hours (Fig 5c). A trend towards fewer monocytes was also observed, but did not reach significance (Fig 5d). These data indicate that endogenous mCRAMP is not involved in the first phase of neutrophil influx, but is required, following induction, for the second phase neutrophil response to *P. aeruginosa* infection. TNF, IL-6, MIP-2, KC and MCP-1 were all highly expressed in response to infection in *Camp −/−* mice at 6 hours (Fig 6a–e), and resolving by 24 hours (Fig 6f–j), but were not significantly different from the responses quantified in infected wild type mice.

### Therapeutic delivery of LL-37 can restore protection against *P. aeruginosa* infection in cathelicidin deficient mice

**Figure 7:**
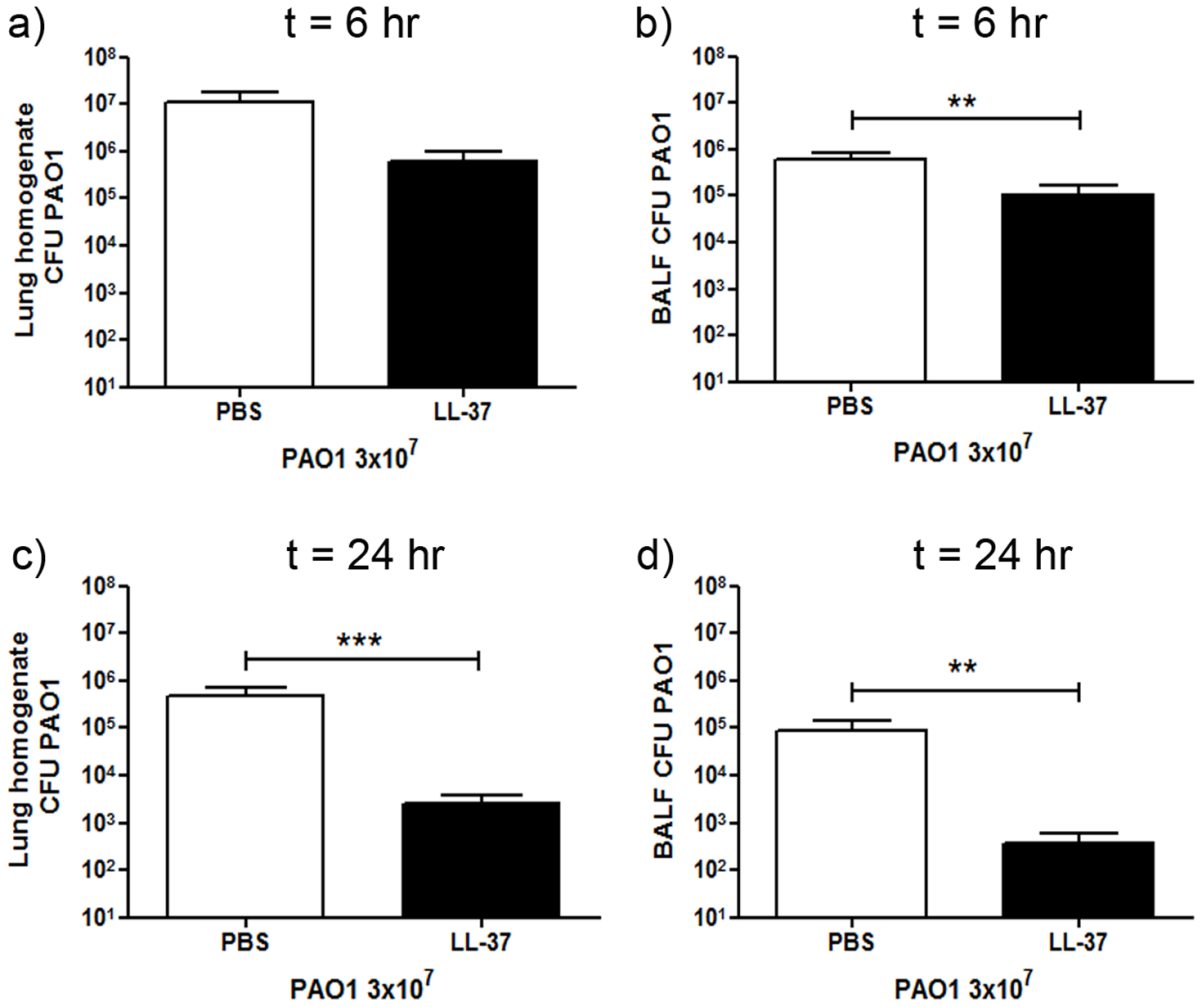
Exogenous LL-37 enhances pulmonary clearance of *P. aeruginosa* in cathelicidin-deficient mice. *Camp−/−* mice were inoculated with 3×10^7^ cfu of *P. aeruginosa* PAO1 or PBS and 10 µg LL-37 peptide or PBS by intranasal delivery. At 6 or 24 hours after inoculation mice were re-weighed and culled, and their lungs were lavaged before homogenisation. BALF and lung homogenates were serially diluted, plated and incubated overnight at 37°C before bacterial colonies were counted and corrected for volume. Mean PAO1 cfu +/− SEM in the lung homogenate (a & c) or BALF (b & d) for infected animals (n≥6 per condition at 6 hours and n≥10 per condition at 24 hours) are displayed. No bacteria were detected in samples from uninfected mice. For statistical analyses bacterial counts were normalised by logarithmic transformation. Analyses were conducted using 2 way ANOVA with Bonferroni's post tests; ** p<0.01, *** p<0.001.

Cathelicidin insufficiency has been associated with increased susceptibility to infection in humans [12], [39], [40]. Thus, in order to determine whether therapeutic use of synthetic human LL-37 could restore cathelicidin-mediated protective antimicrobial function in cathelicidin deficiency, *Camp −/−* mice were infected with *P. aeruginosa* PAO1 *in vivo*, with or without concomitant delivery of LL-37. The profile of weight loss in infected *Camp −/−* mice was not significantly altered by delivery of LL-37 (data not shown). Mice were culled 6 and 24 hours post-infection and the total number of viable bacteria in the BALF (bronchoalveolar lavage fluid) and lung homogenate was assessed (Fig 7 a–d). At 6 hours post-infection, treatment with LL-37 showed no significant effect on the total number of bacteria in the lung homogenates (despite a trend towards enhanced clearance), but led to significantly lower levels of bacteria in the BALF (Fig 7a/b). By 24 hours post-infection, LL-37 treatment had very significantly enhanced pathogen clearance from the lungs, compared to control-treated *Camp −/−* (Fig 7c/d). These data demonstrate that delivery of exogenous synthetic LL-37 can enhance host defence against infection by mechanisms that do not require endogenous host cathelicidin production, and show cross-species functionality of these peptides.

### Therapeutic delivery of LL-37 promotes an early neutrophil response to *P. aeruginosa* infection, associated with enhanced clearance

**Figure 8:**
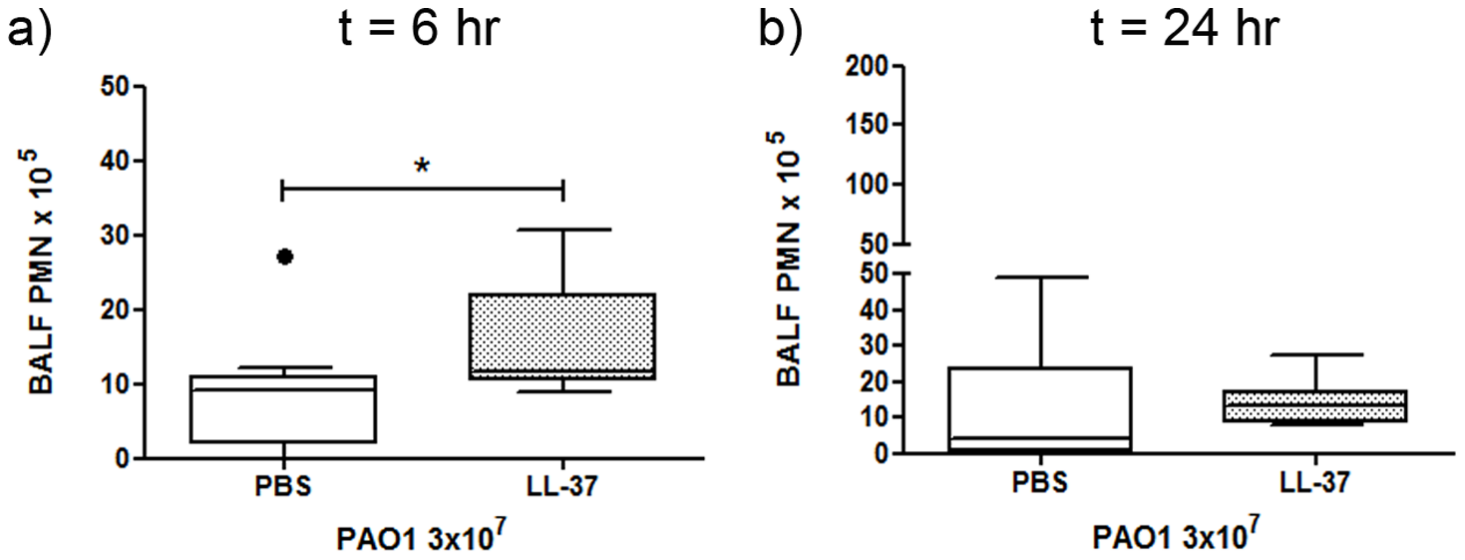
Exogenous LL-37 promotes an early neutrophil response to *P. aeruginosa* in cathelicidin-deficient mice. *Camp−/−* mice were inoculated with 3×10^7^ cfu of *P. aeruginosa* PAO1 and 10 µg LL-37 peptide or PBS by intranasal delivery. At 6 hours (a) or 24 hours (b) after inoculation mice were culled and their lungs were lavaged. BALF was cytocentrifuged and differential counts were conducted for neutrophils. Data show Tukey box and whiskers plots for n≥8 animals per condition. Analyses were conducted using the Mann Whitney test; * p<0.05.

Cathelicidin-mediated enhancement of bacterial clearance was associated with upregulated neutrophil influx in LL-37-treated infected wild type mice (compared to untreated infected controls). However, in addition, endogenous cathelicidin clearly also had a critical role in the induction of a maximal neutrophil responses to infection. Thus, in order to determine whether LL-37-mediated enhanced neutrophil responses were independent of endogenous cathelicidin production, BALF cytospin differential cell counts were also evaluated from LL-37-treated and control infected *Camp −/−* mice at 6 and 24 hours after infection. As also observed in LL-37-treated wild type mice (Fig 2a), early, infection-induced neutrophil influx (at 6 hours) was significantly greater in LL-37-treated mice (Fig 8a), but this early effect of the therapeutic bolus was lost by 24 hours (Fig. 8b). However, whereas wild type mice showed a robust later neutrophil response to infection regardless of peptide treatment (Fig 2c), this second phase neutrophil response failed to occur in infected *Camp −/−* mice, irrespective of peptide treatment (Fig 8b), demonstrating the dependence of this later response upon pathogen-induced *Camp* expression. No significant effects upon monocyte counts were observed (data not shown). These data demonstrate that the early infection-mediated neutrophil response, enhanced by the bolus of LL-37, was independent of endogenous cathelicidin expression and associated with enhanced clearance of pulmonary *P. aeruginosa*.

## Discussion

Cathelicidins are recognised as key multifunctional modulators of innate immunity and host defence against infection, and offer possible novel therapeutic templates. In addition to directly microbicidal potential, these peptides have been described as having a broad range of inflammomodulatory and immunomodulatory properties [2]. However, no clear evidence exists for these functions being involved in cathelicidin-mediated enhanced host defence against pulmonary infection *in vivo*, with the relative significance of microbicidal potential and modulatory functions remaining unclear. Using a murine model of acute *P. aeruginosa* lung infection, we demonstrate cathelicidin-mediated enhancement of bacterial clearance *in vivo* in the absence of direct early microbicidal activity. Administration of synthetic LL-37 promoted an upregulation of the early neutrophil response that was dependent upon both infection and peptide, but was independent of native cathelicidin production, and enhanced bacterial clearance from the lung. Mice deficient in endogenous mCRAMP had a normal initial neutrophil response to infection, during a period in which *Camp* was not initially expressed in the wild type lung. However, these *Camp*−/− mice were deficient in the larger second phase neutrophil response observed in *Camp*-expressing infected wild type mice and had an impaired capacity to clear the infection. To our knowledge, this is the first demonstration of cathelicidins enhancing host defence against infection through primarily modulatory mechanisms *in vivo* and suggests induction of a cathelicidin-mediated protective proinflammatory response.

The associations between hCAP-18/LL-37 expression and susceptibility to infection in humans [39] [40] [11] [12] suggest an important, but as yet undefined role for hCAP-18/LL-37 in innate host defence against infection in humans. Although this peptide has microbicidal potential, its activity is poor against many microorganisms in physiologically relevant environments at the low concentrations found *in vivo* in most systems [27], [28], [29]. However, cathelicidins have additionally been shown to have multiple modulatory activities, including chemotactic function [34], [35], [36], [37], [38], the ability to modulate chemokine, cytokine and cellular responses [30], [41], [42], [43], the capacity to alter leukocyte differentiation and function [44], [45], [46] and cell death modulating properties [28], [47], [48]. Critical to understanding these peptides and utilising their properties therapeutically is the need to clarify their modes of action *in vivo* in infectious contexts.

In this study, an acute murine pulmonary infection model with *P. aeruginosa* was utilised in order to evaluate the capacity of cathelicidins to enhance host defence against infection with a microbe which is largely resistant to these peptides under physiological conditions *in vitro* [27], [28], [29]. Under favourable *in vitro* conditions in which microbicidal properties are evident for LL-37, this peptide has been shown to permeabilise bacterial membranes within minutes [49]. However, we found no evidence for direct microbicidal activity against *P. aeruginosa* after co-incubation with LL-37 *in vivo*, yet exogenously delivered LL-37 was found to significantly enhance pathogen clearance over 24 hours. Although we cannot exclude some alternative form of late direct microbicidal activity of LL-37, even by 6 hours after infection no significant impact on bacterial load of the whole lung could be demonstrated in response to LL-37 treatment, although interestingly a therapeutic bolus of peptide was found to diminish the number of live bacteria accessible to bronchoalveolar lavage at this time point. The reason for this is unclear, but may relate to early removal of the most accessible bacteria by the enhanced neutrophil influx observed. A previous study using adenoviral vectors carrying the cDNA for hCAP18/LL-37, to overexpress the human cathelicidin in the murine lung over the 5 days prior to infection, resulted in the promotion of a small, but significant enhancement of *P. aeruginosa* clearance from the murine lung over a 24 hour period [21]. This was observed to be accompanied by decreased pulmonary TNF levels, but the mechanism underpinning this therapeutic effect was not evaluated and was assumed to be microbicidal. In contrast, we found no evidence to support a microbicidal effect, but demonstrate a peptide-mediated enhanced early neutrophil influx *in vivo*.

Prior research has demonstrated the capacity for cathelicidins to have direct chemotactic activity for human neutrophils and monocytes and murine leukocytes *in vitro* [34], [36] and for murine leukocytes in an experimentally-formed murine air pouch model [36]. In that *in vivo* model, injection of 2 µM LL-37 or mCRAMP into the air pouch significantly enhanced the influx of neutrophils and monocytes within a 4 hour period. This is in contrast to the complete absence of neutrophils observed in our studies in the murine lung 6 hours after instillation of LL-37 alone (Figure 2b). In addition, LL-37 was not found to mediate any significant effects on the number of monocytes in the BALF, in contrast to the previously published findings in other systems [35], [36]. A small, but significant neutrophil response was observed in the lungs of LL-37-treated uninfected mice at 24 hours after instillation (Figure 2d), demonstrating some LL-37-mediated neutrophil influx. However, indirect effects cannot be excluded and LL-37 has also been shown to enhance the production of neutrophil chemokines by other cells [30], [50]. Nevertheless, despite this absence of any substantial response to LL-37 alone, a significantly enhanced pulmonary neutrophilia was observed in response to LL-37 upon concomitant infection (Figure 2a). With regard to this apparent contradiction to previous findings, it is worth noting that even the control air pouches in the previous report yielded a substantial number of neutrophils [36], indicating that this was already an inflamed environment and may in fact be analogous to infected lungs in our study. The requirement for concomitant infection in order to establish the early enhanced LL-37-mediated neutrophil response suggests a synergy with inflammatory mediators that remain to be identified, with no peptide-mediated modulation having been observed in the levels of the chemokines studied (including KC, MIP2, MCP-1, TNF and IL-6). Nevertheless, these data provide clear evidence for the *in vivo* capacity of exogenous LL-37 to modulate the innate cellular immune response in the context of pulmonary infection, enhancing pathogen clearance in the absence of microbicidal activity and having potential therapeutic implications.

In addition to the potential therapeutic roles of exogenous cathelicidins, the primary roles of endogenous peptides in pulmonary infection remain unclear. Studies using *Camp−/−* mice have demonstrated a deficiency in the clearance of both pulmonary *Klebsiella pneumoniae* and *P. aeruginosa* infections [19], [20]. *K. pneumoniae* promoted a later induction of pulmonary *Camp* expression than *P. aeruginosa* and mCRAMP appeared to have a more potent effect on *K. pneumoniae*, with *Camp−/−* mice having a significant and severe clearance defect at 24 hours, resulting in more florid inflammation by 48 hours in the absence of endogenous cathelicidin and increased mortality [20]. Given that, in the case of *K. pneumoniae*, mCRAMP was reported to have microbicidal effects at a relatively modest 1 µM, it is possible that the phenotype in this particular infection was influenced by loss of a relevant microbicidal agent in the face of a lethal infectious dose. Indeed, the late induction of *Camp* in wild type mice infected with *K. pneumoniae* may suggest less relevance for the peptide in the inflammatory response to this particular infection. In contrast, we demonstrate that *P. aeruginosa* infection can ultimately be controlled and cleared even in the absence of mCRAMP, and the earlier expression in wild type mice may indicate a more important role in the inflammatory response. The previous study reported that *Camp−/−* mice infected with *P. aeruginosa* (at a lower infectious dose than in our study) has a significantly impaired bacterial clearance at 48 hours, with a decreased neutrophil response at 24 hrs [20]. These observations are compatible with our study, but were attributed to a loss of a direct chemotactic response to endogenous cathelicidin. Our new data, examining earlier timepoints, indicate that the initial murine pulmonary neutrophil response to *P. aeruginosa* infection precedes induction of and is independent of mCRAMP, and thus proceeded normally in *Camp−/−* mice. However, second phase neutrophil influx was dependent upon mCRAMP expression, which may synergise with infection-induced factors as yet unidentified, and was therefore defective in *Camp−/−* mice, in whom impaired pathogen clearance then occurred. These data suggest that in individuals with impaired endogenous cathelicidin production, an effective, protective pulmonary inflammatory response will be suboptimal.

hCAP-18/LL-37 in humans is pre-formed in neutrophil granules, but can also be induced in a vitamin-D dependent manner in epithelial cells and macrophages [51], [52], [53], [54]. In addition, strategies to induce LL-37 expression are under development, including the use of compounds such as 4-phenylbutyrate (reviewed in [55]), which can effectively upregulate hCAP-18/LL-37 expression *in vitro*, including in airway epithelial cells [56], and *in vivo* in a model of Shigella infection [57]. Such approaches may be of value in enhancing protective cathelicidin expression in humans, particularly in vitamin-D insufficient seasonal conditions. However, it was also important to consider whether therapeutic application of cathelicidin could provide rapid short term improvement of host defence in the absence of effective endogenous cathelicidin expression. In this regard, our studies demonstrate that the delivery of LL-37 to *P. aeruginosa* infected mice could promote an early neutrophil response and enhanced pathogen clearance in *Camp−/−* mice as effectively as in wild type mice. These data indicate that this protective effect was in response to the exogenous LL-37 delivered, independent of endogenous mCRAMP expression and of native cathelicidin release from incoming PMN and supports the potential for the use of exogenous peptides in infection.

Thus, using a murine model of acute *P. aeruginosa* lung infection, we demonstrate cathelicidin-mediated enhancement of bacterial clearance *in vivo* in the absence of direct microbicidal activity. The delivery of exogenous cathelicidin functioned to enhance a protective pro-inflammatory response to infection, promoting bacterial clearance from the lung, with an infection- and peptide-dependent early neutrophil response that was independent of native cathelicidin production. Furthermore, although *Camp*−/− mice had an intact early cellular inflammatory response (which was comparable to cathelicidin-sufficient animals in the period preceding the induction of mCRAMP expression), they had significantly impaired bacterial clearance and absence of a second phase neutrophil response to infection. These finding demonstrate the importance of the inflammomodulatory properties of cathelicidins in pulmonary infection *in vivo* and highlight the significance of understanding and utilising these properties in the development of novel therapeutic approaches.
